# Intravesical BCG in bladder cancer induces innate immune responses against SARS-CoV-2

**DOI:** 10.3389/fimmu.2023.1202157

**Published:** 2023-07-13

**Authors:** Renate Pichler, Gabriel Diem, Hubert Hackl, Jiří Koutník, Laura S. Mertens, David D`Andrea, Benjamin Pradere, Francesco Soria, Andrea Mari, Ekaterina Laukhtina, Wojciech Krajewski, Jeremy Yuen-Chun Teoh, Francesco Del Guidice, Marco Moschini, Martin Thurnher, Wilfried Posch

**Affiliations:** ^1^ Department of Urology, Comprehensive Cancer Center Innsbruck, Medical University of Innsbruck, Innsbruck, Austria; ^2^ Institute of Hygiene and Medical Microbiology, Medical University of Innsbruck, Innsbruck, Austria; ^3^ Institute of Bioinformatics, Biocenter, Medical University of Innsbruck, Innsbruck, Austria; ^4^ Institute of Cell Genetics, Medical University of Innsbruck, Innsbruck, Austria; ^5^ Department of Urology, The Netherlands Cancer Institute - Antoni van Leeuwenhoek Hospital, Amsterdam, Netherlands; ^6^ Department of Urology, Medical University of Vienna, Vienna, Austria; ^7^ Department of Urology, Croix Du Sud Hospital, Quint-Fonsegrives, France; ^8^ Department of Urology, Molinette Hospital, University of Turin, Turin, Italy; ^9^ Department of Experimental and Clinical Medicine, University of Florence - Unit of Oncologic Minimally-Invasive Urology and Andrology, Careggi Hospital, Florence, Italy; ^10^ Institute for Urology and Reproductive Health, Sechenov University, Moscow, Russia; ^11^ Department of Minimally Invasive and Robotic Urology, Wrocław Medical University, Wroclaw, Poland; ^12^ Department of Surgery, S.H. Ho Urology Centre, The Chinese University of Hong Kong, Hong Kong, Hong Kong SAR, China; ^13^ Department of Maternal Infant and Urologic Sciences, ‘Sapienza’ University of Rome, Policlinico Umberto I Hospital, Rome, Italy; ^14^ Department of Urology, IRCCS Ospedale San Raffaele and Vita-Salute San Raffaele University, Milan, Italy; ^15^ Immunotherapy Unit, Department of Urology, Medical University of Innsbruck, Innsbruck, Austria

**Keywords:** BCG, bladder cancer, COVID-19, SARS-CoV-2, trained immunity, viral infections

## Abstract

BCG is the most efficient adjuvant therapy for high-risk, non-muscle-invasive bladder cancer (NMIBC). Both innate and adaptive immune responses have been implicated in BCG-mediated effects. BCG vaccination can boost innate immune responses via trained immunity (TI), resulting in an increased resistance to respiratory viral infections. Here we evaluated for the first time whether intravesical application of BCG triggers increased immunity against SARS-CoV-2 in patients with high-risk NMIBC. Serum and peripheral blood mononuclear cells (PBMCs) from heparinized whole blood samples of 11 unvaccinated SARS-CoV-2-naïve high-risk NMIBC patients were collected at baseline and during BCG treatment in a pre-COVID-19 era. To examine B-cell or T cell-dependent adaptive immunity against SARS-CoV-2, sera were tested for the presence of SARS-CoV-2 neutralizing antibodies. Using a SARS-CoV-2 peptide pool, virus-specific T cells were quantified via IFNγ ELISpot assays. To analyze innate immune responses, mRNA and protein expression levels of pro- and anti-inflammatory cytokines were measured after a 24-hour stimulation of PBMCs with either BCG or SARS-CoV-2 wildtype. ATAC- sequencing was performed to identify a potential epigenetic reprogramming in immune cells. We neither identified SARS-CoV-2 neutralizing antibodies nor SARS-CoV-2- reactive T cells, indicating that intravesical BCG did not induce adaptive immunity against SARS-CoV-2. However, a significant increase in mRNA as well as protein expression of IL-1β, IL-6 and TNFα, which are key cytokines of trained immunity, could be observed after at least four intravesical BCG instillations. Genomic regions in the proximity of TI genes (*TLR2*, *IGF1R*, *AKT1, MTOR, MAPK14, HSP90AA1*) were more accessible during BCG compared to baseline. Although intravesical BCG did not induce adaptive immune responses, repetitive intravesical instillations of BCG induced circulating innate immune cells that produce TI cytokines also in response to SARS-CoV-2.

## Introduction

Intravesical Bacillus Calmette-Guérin (BCG) immunotherapy has been considered the most successful adjuvant treatment in preventing recurrence and progression of high-risk non- muscle-invasive bladder cancer (NMIBC) for more than 40 years (1, 2). Urology practices have also been affected by the global COVID-19 pandemic (3, 4). Clinical adjustments in the use of BCG schedule in high-risk NMIBC were the consequence of the COVID-19 pandemic. In detail, BCG maintenance ongoing for longer than 1 year could be safely terminated for high-risk NMIBC patients, according to the EAU´s COVID-19 recommendations to protect both patients and healthcare workers (3, 4).

The protective effect of BCG against several viral respiratory infections is mediated via a process termed trained immunity (TI). TI comprises epigenetic as well as metabolic reprogramming of innate immune cells, facilitating the enhanced production of the pro- inflammatory TI cytokines IL-1β, IL-6 and TNFα, when challenged with a secondary irrelevant bacterial or viral stimulus (5, 6). Through TI, BCG vaccination can boost innate immune responses, resulting in reduced viremia, increased TI cytokine production and faster viral elimination (5, 7). Consequently, the development of TI is desirable to generate protection against COVID-19. First data already indicate that BCG might also provide protection against COVID-19 infection (7–9). However, these observational reports cannot confirm a causal association between BCG vaccination and decreased COVID-19 infection as well as mortality rate. Thus, various randomized controlled clinical trials are still ongoing (NCT04384549, NCT04659941, NCT04461379) to determine whether BCG vaccine can prevent COVID-19 infection and severity, especially in healthcare workers or elderly people (10).

When focusing on bladder cancer, the repetitive intravesical BCG application induces a local immune response that ultimately translates into TI. Consequently, intravesical BCG has not only a local but also systemic effects. Recently, BCG-induced TI has been shown to significantly decrease the risk for respiratory viral infections in NMIBC patients (11, 12). However, a possible protective effect also against SARS-CoV-2 through intravesical BCG-induced TI in NMIBC has not been shown to date. We hypothesized that boosting innate immune cells by intravesical BCG instillations with induction of TI could also induce innate immunity against SARS-CoV-2 in NMIBC patients. Therefore, the objective of this study was to analyze, for the first time, the innate and adaptive immune responses against SARS-CoV-2 wildtype in blood samples of patients with (very) high-risk NMIBC who underwent intravesical BCG treatment in the pre-COVID-19 era.

## Materials and methods

### Patients

To rule out preexisting immunity to SARS-CoV-2 either by vaccination or infection, we used blood samples from our biobank of consecutive patients with the diagnosis of a primary or recurrent (very) high-risk papillary NMIBC (Ta high-grade or T1) with or without concomitant carcinoma *in situ* (CIS) of the bladder during a pre-COVID-19 era (2014-2015). The SARS-CoV- 2-naïve status was confirmed in all patients by testing cellular and humoral adaptive immunity (neutralization assay and IFNγ ELISpot). All patients were free of visible papillary tumor at the start of BCG induction as determined via second TURB (except primary, isolated CIS) or negative cystoscopy and/or cytology at most 4 weeks before start of BCG therapy. The study was approved by the local ethical committee of the Medical University of Innsbruck (study number AN2014-0121; 336/4.3).

### Interventions, follow-up and blood sample collection

**Figure 1:**
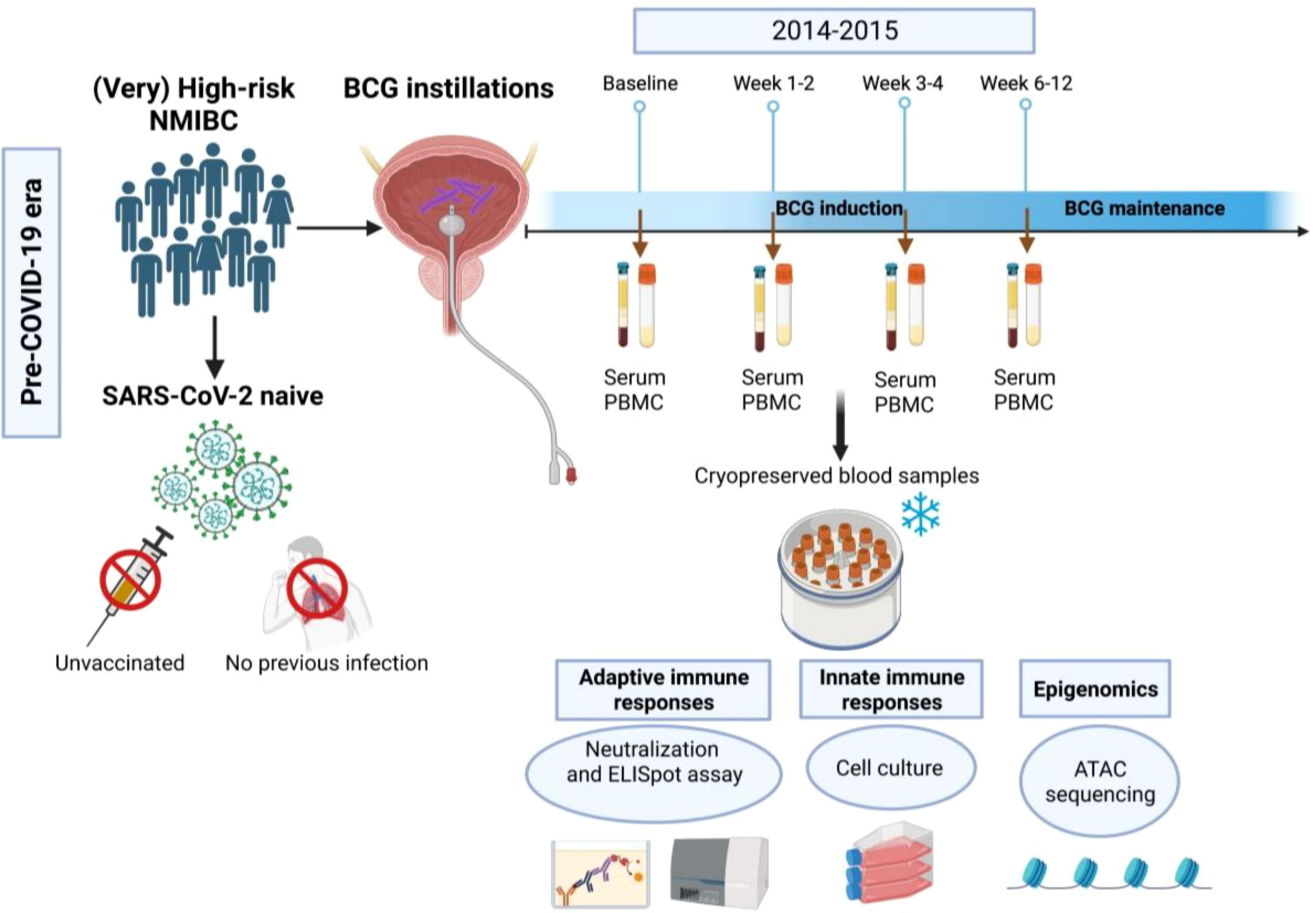
Flow chart of the study. In this study we used blood samples that have been collected from (very) high-risk NMIBC patients undergoing intravesical BCG therapy in the pre- COVID-19 era (2014-2015) using four time points (baseline, 1-2 weeks, 3-4 weeks and 6-12 weeks during BCG).

The BCG treatment schedule at the Department of Urology of the Medical University of Innsbruck was based on a standard regimen of a 6-week induction course followed by 3- weekly maintenance courses at 3, 6, 12, 18, 24, 30, and 36 months according to the current EAU guidelines (1). Each instillation contained 2×10^8^ - 3×10^9^ viable units from live attenuated BCG bacteria strain seed RIVM derived from seed 1173-P2 (BCG Medac, Wedel, Germany). Follow-up examinations were performed according to institutional practice including cystoscopy and cytology (voided urine as well as bladder washing) 3-monthly for 2 years and every 6 months thereafter until 5 years, and then yearly. Upper urinary tract imaging (CT urography) was performed once a year or in case of recurrence (1). Blood sample collection was performed in the pre-COVID-19 era (2014-2015) as described previously (13). In brief, serum and heparinized whole blood (40-50 mL in EDTA tubes) was collected before each BCG bladder instillation. Peripheral blood mononuclear cells (PBMCs) were prepared from heparinized whole blood by Ficoll density centrifugation and aliquots (5×10^6^ cells) were cryopreserved in liquid nitrogen. Serum samples were obtained under standard conditions, clotted at 4–8°C and then centrifuged at 3200 rpm for 6 min. Aliquots of 1.8 ml were stored at −80°C (13). For this follow-up study, we selected 4 samples at 4 different time points for each patient: baseline (before

the first BCG induction) and during BCG (1-2 weeks/early, 3-4 weeks/mid and 6-12 weeks time interval/late) treatment. The study flow chart is presented in **Figure 1**.

### Determination of SARS-CoV-2 RBD-specific antibody titers

Sera from BCG treated patients as well as untreated but SARS-CoV-2 recovered individuals were analyzed for SARS-CoV-2 RBD-specific antibodies using SARS-CoV-2 IgG II Quant Assay (Abbott, USA). Results from this chemiluminescent microparticle immunoassay (CMIA) were calculated to BAU/ml and the cut-off value for positive results was defined at 7.1 BAU/ml according to manufacturer instructions.

### Virus neutralization assay

Serum was serially diluted (1:8 – 1:2048) and incubated with replication competent SARS-CoV-2 wildtype virus (SARS-CoV-2 USA/WA1/2020, 2.5x10^2^ PFU/ml) for 1h at 37°C as described previously by our group (14). The dilutions were used as inoculum on Vero- TMPRSS2-ACE2 cells for 1h at 37°C, 5% CO2. After medium exchange, cells were further cultivated for 16h. Subsequently, plaque forming units (PFU) were measured after fixation and permeabilization using immunofluorescently labelled antibodies targeting SARS-CoV-2 nucleocapsid. Imaging and counting were performed using an ImmunoSpot analyser (Cellular Technology Limited, OH, USA).

### SARS-CoV-2 or BCG-specific T cell response (IFNγ ELISpot)

PBMCs (0.5x10^5^) were treated with CEF/CEFTA (1µg/ml each) as positive control, SARS-CoV-2 peptide pool (spike, matrix and nucleocapsid proteins, Mabtech, Sweden) or a mixture of *Mycobacterium tuberculosis* peptides for 24h as previously described (15, 16). The CEF peptide pool consists of 23 MHC class I-restricted viral peptides from human CMV, EBV, and influenza virus induces secretion cytokine secretion from antigen-specific human CD8 T cells. The CEFTA peptide pool consists of 35 MHC class II-restricted peptides from human CMV, EBV, influenza virus, tetanus toxin, and adenovirus 5 to induce cytokine secretion of specific CD4 T cells. The SARS-CoV-2 peptide pools is a mixture of 100 peptides from the viral spike, membrane and nucleocapsid respectively and used at a concentration of 1 µg/ml each. The *Mycobacterium tuberculosis* peptide pool consists of a mixture of peptides pools from the antigenic targets ESAT-6, CFP-10 and EspC (1µg/ml each). After IFNγ detection, spots were imaged and counted using an ImmunoSpot analyser (Cellular Technology Limited, OH, USA).

### *In vitro* induction of trained immunity

PBMCs (3 x 106) from three healthy donors were isolated using Ficoll isolation and primed for 24h with LPS (100 ng/ml), BCG (MOI 0.1) or left untreated (unprimed). Cells were then incubated at 37°C and 5% CO2 for 5 days before a 24h re-stimulation with LPS (100 ng/ml), BCG (MOI 0.1), SARS-CoV-2 (MOI 0.1) or mock treatment. Trained immunity was assessed by measuring the IL-1β mRNA expression levels in each condition and calculation of the delta delta CT as previously described by Livak et al. (17), relative to the unprimed and untreated cells.

### Cell culture and cytokine analysis

Frozen PBMC stocks of each donor and each time point were thawed and cultured in RPMI- 1640 medium supplemented with 10% FCS and 1% L-glutamine for 2-3h before treatment (all reagents were obtained from Sigma Aldrich, MO, USA). Cells were then either left untreated, stimulated with LPS (*E. coli* O26:B6, 100 ng/ml, Sigma Aldrich, St. Louis, MO, USA) a live attenuated BCG strain RIVM (BCG Medac, Wedel, Germany; MOI: 1) or replication competent SARS-CoV-2 wildtype virus (SARS-CoV-2 USA/WA1/2020; MOI: 0.1) for 24h. For quantification of cytokine mRNA expression PBMCs were harvested and lysed 24h after treatment and RNA was extracted using a commercially available kit (Dr. P Kit, BioChain, CA, USA) following cDNA synthesis (LunaScript RT Supermix, New England Biolabs, MA, USA). Expression of IL-1β, IL-6, TNFα, IL-12A, IL-18, IFNγ and IL-10 mRNA was analyzed by PrimePCR Assays via multiplex qPCR using iQ Multiplex Powermix (Bio-Rad laboratories, CA; USA). Relative quantification of target genes was performed using the delta-delta CT method as previously described and normalized to the respective untreated controls (18). Cytokine protein concentrations of IL-1β, IL-6, IL-10, IL-18, TNFα and IFNγ in cell culture supernatants 24h post stimulation were measured using Bio-Plex systems (Bio-Rad Laboratories, CA, USA) according to the manufacturers’ instructions.

### Next generation sequencing

Assay for transposase accessible chromatin (ATAC), including tagmentation, library preparation and sequencing were performed by Genewiz (Azenta Life Sciences, MA, USA) on PBMCs from two BCG-treated NMIBC patients (responder) at baseline and during BCG (mid) as well as from two healthy donors. Raw data were preprocessed including mapping to the hg38 reference genome using bowtie2 and peak calling by MACS2. Differentially accessible regions in proximity to annotated genes including TI genes (TIDB) between the two time points (during BCG versus baseline) were identified using the R packages csaw and edgeR. We only focused on consensus regions with sufficient normalized counts (logCPM>1), which were more than three-fold enriched and annotated in the promoter or intron of coding genes excluding X, Y chromosomes and blacklisted regions. Overrepresentation analysis of biological processes (GO) and pathways (Reactome) were performed using DAVID, ConsensusPathDB, and ClueGO (**Supplementary dataset S1**).

### Statistical analysis

All statistical analyses were performed on GraphPad Prism 9 (GraphPad Software Inc., San Diego, CA, USA). Paired t-test was used for neutralization assays comparing patients’ sera before and after BCG treatment or Mann-Whitney test for SARS-CoV-2 vaccinated/unvaccinated individuals. ELISpot data were analyzed via 2-way ANOVA with Šidák’s correction for multiple comparisons. For cytokine analysis, non-parametric Kruskal Wallis test with Dunn’s correction for multiple comparisons was applied. A *p*-value of < 0.05 was considered significant. Graphics were produced with BioRender (www.biorender.com).

## Results

Dynamic blood samples of eleven high-risk NMIBC patients during intravesical BCG treatment were available for further analysis. Descriptive patient and tumor characteristics are shown in **Table 1**. All patients were classified as BCG responders during a mean (range) follow-up of 41 (18-50) months, respectively.

**Table 1:** Clinicopathologic features of (very) high-risk NMIBC patients treated with intravesical BCG therapy (n=11).

**Age, mean (SD), *years***	65.3 (6.7)
Sex, n (%)	
female	2 (18.2%)
male	9 (81.8%)
Smoking status, n (%)	
never smoker	1 (9.1%)
former smoker	3 (27.3%)
current smoker	7 (63.6%)
History of BCa, n (%)	
primary NMIBC	9 (81.8%)
recurrent NMIBC	2 (18.2%)
Tumor diameter, n (%)	
< 3 cm	8 (72.7%)
≥ 3 cm	3 (27.3%)
Number of tumors, n (%)	
1	7 (63.6%)
2-7	4 (36.4%)
> 7	–
pT stage, n (%)	
Ta	3 (27.3%)
T1	8 (72.7%)
Tumor grade (WHO 2004/2016), n (%)	
LG	4 (36.4%)
HG	7 (63.6%)
Tumor grade (WHO 1973), n (%)	
G1	2 (18.2%)
G2	2 (18.2%)
G3	7 (63.6%)
**Concomitant CIS, n (%)**	6 (54.5%)

CIS, carcinoma in situ; LG, low grade; HG, high grade; BCa, bladder cancer; NMIBC, non-muscleinvasive bladder cancer.

**Figure 2:**
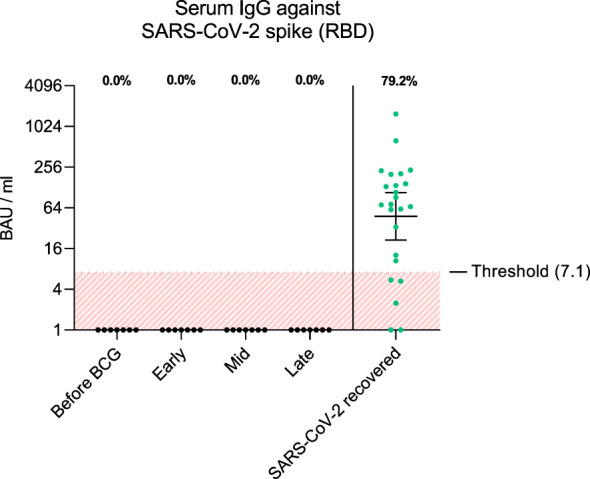
The graph shows antibody titers against wildtype SARS-CoV-2 spike RBD in BAU/ml (binding antibody units per ml) from each patient and time interval (black dots). As comparison, antibody titers from SARS-CoV-2 recovered individuals 1 month post infection are shown (green dots, n=24). Fraction of positive titers are indicated as percentages above each group. Titers >7.1 BAU/ml were defined as positive according to the manufacturer’s instructions. Time points: 1-2 weeks/early, 3-4 weeks/mid and 6-12 week-interval/late during BCG. Data is presented as geometric mean (horizontal line) with 95% confidence interval (whiskers).

**Figure 3:**
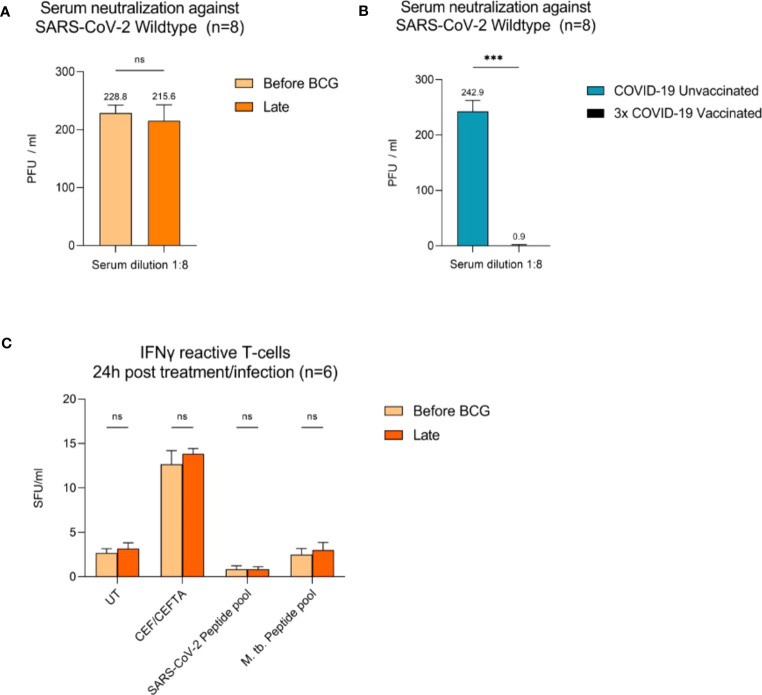
Virus neutralization assays and SARS-CoV-2/BCG specific IFNγ T cell responses. **(A)** Serum neutralization against SARS-CoV-2 wildtype in eight BCG-treated high-risk NMIBC patients (baseline and during BCG). **(B)** Serum neutralization in the internal control group (COVID-19 unvaccinated and 3x vaccinated patients, n=8). **(C)** IFNγ reactive T cells 24h post BCG/SARS-CoV-2 peptide pool (n=6). PBMCs (0.5x10^5^) were treated with CEF/CEFTA as positive control. Data represent mean with SD; *p <.05, **p <.01; ***p <.001.

To examine adaptive immune responses, we analyzed plasma antibodies against SARS-CoV-2 RBD and found no positive titers in all tested patients regardless of BCG instillation time interval, **Figure 2**. Next, we performed virus neutralization and IFNγ ELISpot assays. We found no cross-reactive antibodies against SARS-CoV-2 wildtype in BCG-treated NMIBC patients at baseline and during BCG therapy (mean, 228.8 *vs.* 215.6 PFU/mL; *p*=0.266), **Figure 3A**. As a control, we performed neutralization assays also in healthy, non-NMIBC blood donors who were either COVID-19 unvaccinated (n=8) or 3x COVID-19 vaccinated (n=8). Neutralizing antibodies could only be detected in vaccinated individuals (mean, unvaccinated *vs*. 3x vaccinated: 242.9 *vs.* 0.9 PFU/mL; *p*<0.0001), **Figure 3B**. Furthermore, to evaluate a potential cross-reactive T cell induction, we challenged T cells with either SARS-CoV-2 wildtype-specific or BCG-specific peptide pools as well as CEF/CEFTA as positive control for 24h and measured the presence of pathogen-specific T cells via IFNγ ELISpot assays. We could not detect SARS-CoV-2 reactive T cells in our patients, **Figure 3C**.

**Figure 4:**
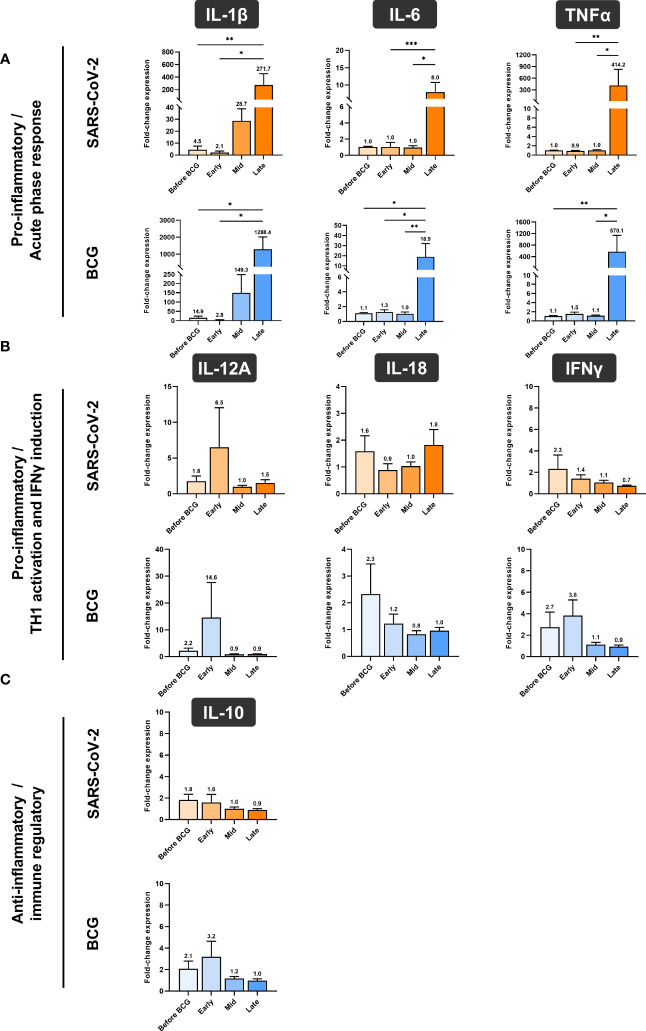
Relative quantification of cytokine mRNA expression by multiplex qPCR. **(A)** Pro- inflammatory cytokines IL-1β, IL-6 and TNFα, **(B)** cytokines associated with Th1 response IL- 12A, IL-18 and IFNγ and **(C)** anti-inflammatory cytokine IL-10 after a 24h stimulation of PBMCs with either BCG or SARS-CoV-2 wildtype are shown. PBMCs were taken before the start of BCG induction (baseline) and at early, mid and late time intervals during BCG treatment. Data is presented as fold-change expression relative to untreated control group (mean with SEM and mean values annotated above each individual bar; *p <.05, **p <.01; ***p <.001). Time points: baseline (before BCG induction), 1-2 weeks/early, 3-4 weeks/mid and 6-12 week-interval/late during BCG.

Next, we investigated whether BCG instillations result in altered innate immune responses in patients with NMIBC. After a 24h stimulation of PBMCs with either BCG or SARS-CoV-2 wildtype, cytokine mRNA and protein expression levels were measured via multiplex qPCR. We found a significant increase in mRNA expression of the pro-inflammatory cytokines IL-1β, IL-6 and TNFα in response to BCG stimulation relative to the respective untreated controls, **Figure 4A**, lower panel, blue bars. The highest peak of these cytokines was detected after at least four BCG instillations (late time interval), while no response was detected during the early or mid-time intervals, **Figure 4A**. A very similar cytokine profile was observed after 24h exposure with SARS-CoV-2 wildtype, **Figure 4A**, upper panel, orange bars. No change in IL-12A, IL-18, IFNγ and IL-10 mRNA expression was identified in response to stimulation with BCG or SARS-CoV-2 wildtype relative to untreated, **Figures 4B, C**.

**Figure 5:**
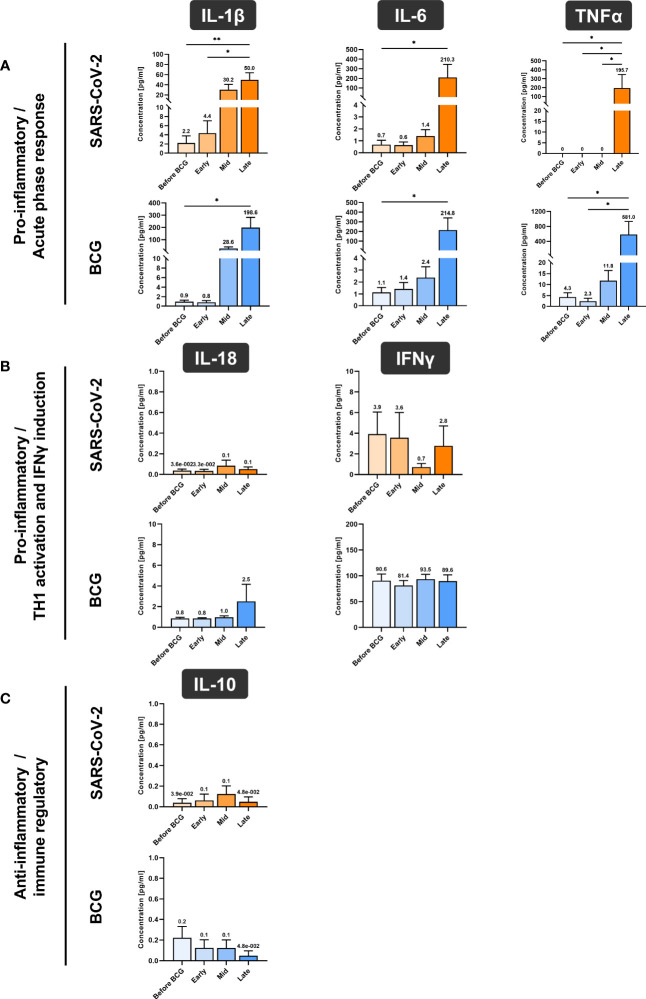
Cytokine quantification using Luminex xMAP technology. **(A)** Pro-inflammatory cytokines IL-1β, IL-6 and TNFα, **(B)** cytokines associated with Th1 response IL-18 and IFNγ and **(C)** anti-inflammatory cytokine IL-10 were measured in cell culture supernatant of PMBCs after 24h stimulation with either BCG or SARS-CoV-2 wildtype are presented. PBMCs were prepared before the start of BCG induction (baseline) and at early, mid and late time intervals during BCG treatment. Data represent means with SEM and mean values annotated above each individual bar (*p <.05, **p <.01; ***p <.001). Time points: baseline (before BCG induction), 1-2 weeks/early, 3-4 weeks/mid and 6-12 week-interval/late during BCG. IL-12A data are not shown as their levels were not detectable and samples for repetition were no more available.

**Figure 6:**
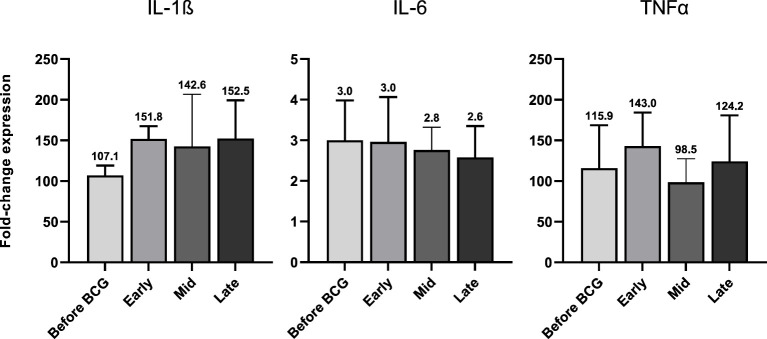
IL-1β, IL-6 and TNFα mRNA expression after 24h of LPS (100 ng/ml) treated PBMCs from patients before and during various time-points of BCG treatment. Data is presented as foldchange expression relative to untreated control group (mean with SEM). Time points: 1-2 weeks/early, 3-4 weeks/mid and 6-12 week-interval/late during BCG.

In accordance with the increase of mRNA expression, we also found significantly elevated secretion of IL-1β but also of IL-6 and TNFα protein after at least four BCG instillations (late time interval), **Figure 5A**. Importantly, these cytokines are considered the key markers of trained immunity. However, expression of the Th1 cytokines IL-18 and IFNγ as well as the anti-inflammatory cytokine IL-10 did not change, **Figures 5B, 5C**. To test if these changes are specific to the BCG or SARS-CoV-2 stimulus, we included a bacterial LPS treatment as potent activator of innate immune cells and observed a strong induction of the pro-inflammatory cytokines IL-1β, IL-6 and TNFα after 24h, independent of patient or instillation time interval, **Figure 6**.

**Figure 7:**
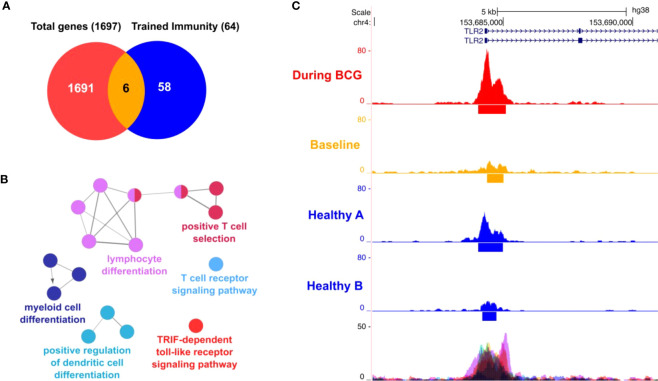
ATAC-seq analysis. **(A)** Venn diagram showing 6 genes (*AKT1, HSP90AA1, IGF1R, MAPK14, MTOR, TLR2*) overlapping between 64 trained immunity genes and 1697 genes with differentially accessible regions in their promoter or introns. **(B)** Differential affected immune system related processes (during BCG versus baseline) using ClueGO **(C)** Representative sequencing tracks for the TLR2 locus showing significantly higher ATAC-seq peaks at the promoter during BCG compared with baseline. Modifications of H3K4me3 were shown at the same region near the transcription start site of *TLR2*.

To establish our experimental protocol for assessing trained immunity, we initially investigated the *in vitro* induction of trained immunity as described by Arts et al. (19) with LPS and BCG using PBMCs from fresh, healthy human blood donations. Cells were primed for 24h with LPS (100 ng/ml) or BCG (MOI: 0.1) or left untreated (i.e. unprimed). After 5 days, cells were re-stimulated with the same conditions and additionally with SARS-CoV-2 (MOI 0.1). We measured the IL-1β mRNA expression in these cells and calculated the fold-change compared to the unprimed and untreated condition, **Figure S1**, black bars. We observed a strong induction of IL-1β expression in the unprimed but LPS-treated conditions (**Figure S1**, LPS treated, black bars) which was greatly enhanced (>3 times more) when cells previously primed with LPS, **Figure S1**, LPS treated, pink bars. Similar results were obtained for cells treated with BCG, **Figure S1**, turquoise bars. Here, IL-1β mRNA expression was higher (>5 times more) in cells that were previously primed and stimulated with BCG compared to unprimed cells, **Figure S1**, BCG, turquoise bars. At the same time, in BCG-primed cells we could also observe a stronger cytokine induction in presence of SARS-CoV-2, which is in line with the results using our patient samples. One basis of trained immunity are epigenetic changes to facilitate a rapid and increased innate immune response. To address this aspect, we performed ATAC-seq analysis. Comparing two time points (during BCG vs. baseline), we identified differentially accessible genomic regions in proximity to 1697 genes. Among 64 TI candidate genes, 6 genes were overlapping (*TLR2*, *IGF1R*, *AKT1, MTOR, MAPK14, HSP90AA1)*, **Figure 7A**. We also performed an analysis of the overrepresented immune-related pathways and biological processes of the more accessible genes during BCG compared with baseline. Especially, myeloid differentiation was affected (**Figure 7B**). We particularly assessed an H3K4me3 signal in the identified regions near the TI genes, confirming H3K4me3 modifications at a more accessible region near transcription start site of all 6 genes. Next, as an example we show TLR2, which is one of the two BCG-specific toll-like receptors (TLR), **Figure 7C**.

## Discussion

Despite 40 years of clinical research (2), the exact immune mechanism of BCG-mediated antitumor activity is still not fully established (17). However, activation of a predominant Th1- type immune response by infiltrating effector cells into the bladder wall is essential for a subsequent clinical BCG response (13, 20–22). Accordingly, BCG responders showed a significant increase of Th1-type urinary cytokines during BCG therapy (13, 20, 21). In contrast, BCG was totally ineffective in IFNγ or IL-12 knockout mice in a syngeneic orthotopic model of bladder cancer (23). The Th1-type immune response is elicited by dendritic cells, which are the most potent antigen presenting cells. BCG induces the activation/maturation of dendritic cells (24), and thus initiates the Th1-type immune cascade (20, 21). In summary, intravesical BCG elicits a potent local immune response and recruits effector T cells into the NMIBC tumor microenvironment, suggesting that T lymphocytes are critical to BCG-mediated clinical efficacy resulting in BCG response (25). Our ClueGO analysis, including BCG responders, supports these findings, highlighting a strong alteration in lymphocyte differentiation and T cell response as well.

**Figure 8:**
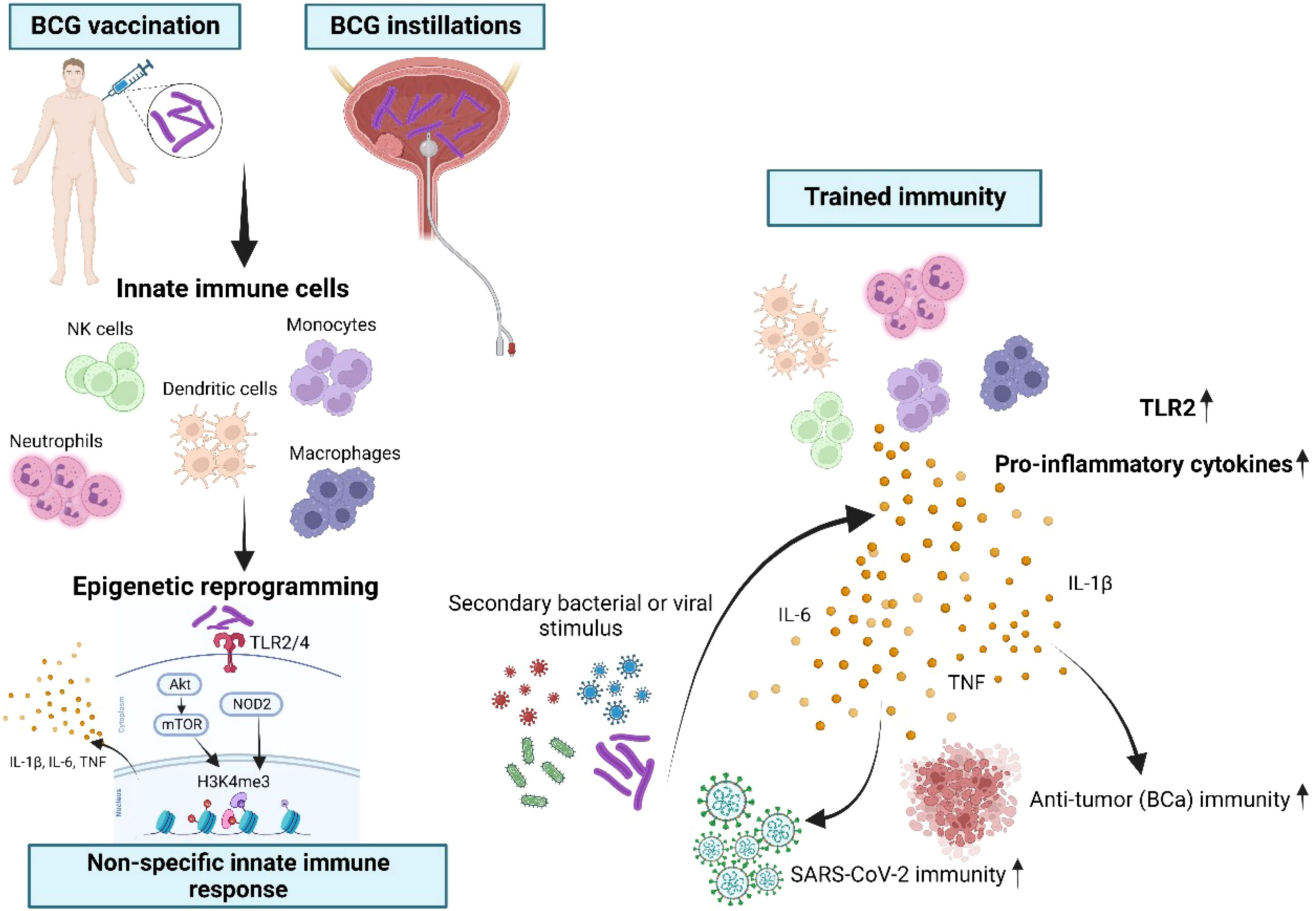
Schematic overview of BCG-induced trained immunity (TI). Innate immune cells such as monocytes, NK cells, dendritic cells, neutrophils, macrophages and their progenitor cells can be trained through repetitive BCG administration, which induces epigenetic and metabolic reprogramming of these cells. BCG is binding to the toll-like receptors (TLR)2 and TLR4 and thus leads to downstream epigenetic modifications at H3K4me3 through both the Akt/mTOR and NOD2 pathways. As a consequence, this specific modification of H3K4me3 facilitates the enhanced production of IL-1β, IL-6 and TNFα, known as the key cytokines of trained immunity (26–28). During a secondary bacterial or viral stimulation trained innate immune cells display an increased capacity to produce pro-inflammatory cytokines, resulting in improved protection also against unrelated pathogens. Importantly, in our study intravesical instillations as a route of BCG administration were suitable to induce TI, reflected by increased production of TI cytokines after at least four BCG instillations at mRNA and protein levels. BCG administration also resulted in epigenetic modifications of TI-related metabolic genes and of the gene encoding TLR2, which is a specific receptor for BCG. Most importantly, we demonstrated for the first time that high-risk NMIBC patients undergoing intravesical BCG showed an increased SARS-CoV-2 innate immune responsiveness indicative of TI.

Activated myeloid cells such as dendritic cells and macrophages can further increase Th1 immune responses by producing cytokines such as IL-1β, IL-6, TNFα, as well as IL-12 and IL-18 after stimulation of toll-like and NOD-like receptors by BCG (26, 27). Myeloid cells can also develop TI following BCG vaccination. TI is characterized by an epigenetic and functional reprogramming, which facilitates the production of the pro-inflammatory cytokines IL-1β, IL- 6 and TNFα, in response to a secondary irrelevant bacterial or viral stimulus (28, 29). Althoughmonocytes, macrophages and NK cells have dominant roles in innate immune memory, other mature innate immune cells, but also hematopoietic stem cells as well as progenitor cells have been implicated in TI (30). BCG is the most potent agent that induces TI through TLR2 and TLR4 activation and downstream signaling via Akt/mTOR and NOD2. Both pathways are required for epigenetic modifications of H3K4me3, thus facilitating an increased pro-inflammatory cytokine production (**Figure 8**) (31). Enhanced neutrophil function through BCG vaccination is associated with genome-wide epigenetic modification of H3K4me3 (32). Moreover, macrophages are trained to increase the expression of various pattern recognition receptors, chemokine receptors and costimulatory and/or signaling molecules that also correlated with modification of H3K4me3 (33). Accordingly, we also demonstrated H3K4me3 modifications at a more accessible region near transcription start site of TI genes such as the BCG receptor *TLR2* induced by BCG instillations. Arts et al. showed that BCG-induced TI was accompanied by a metabolic shift towards glycolysis through activation of the Akt/mTOR pathway (34). Thus, BCG-induced epigenetic and metabolic reprogramming influence each other. This fact means that inhibition of metabolism can reverse epigenetic changes in an *in vitro* TI model, resulting in reduced cytokine responses upon re-stimulation (35).

The repetitive activation of innate immune cells with BCG is the most important driver of enhanced innate immune responsiveness. Along this line, repetitive BCG administration has been shown to decrease the susceptibility to respiratory viral infections with respiratory syncytial virus, influenza A virus and herpes simplex virus type 2 (36, 37). This prompted us to test whether intravesical BCG might also induce TI-like innate immunity against SARS-CoV-2 in NMIBC. Several observational studies have already attempted to show a possible link between intravesical BCG instillations and COVID-19 infections (5, 38–40). No differences in the incidence of COVID-19 infections in BCG-treated NMIBC patients could be shown. However, these findings must be interpreted with caution because they did not examine causal and mechanistic associations between intravesical BCG application and immunity against SARS-CoV-2 (5, 38–40). With regard to severity of the disease, we believe that BCG-induced TI may indeed help prevent detrimental hyperinflammation associated with viremia. Severe cases of Covid-19 tend to have lower lymphocyte counts (lymphopenia) (41). Intriguingly, a recent study also assessed short-term effects of BCG on innate immune responses during the BCG induction regimen and found that one week after the first BCG instillation the total number of white blood cells in the circulation was significantly increased compared to pre-BCG-1. Obviously, BCG treatment also appears appropriate to counteract lymphopenia and may thus improve the clinical course of Covid-19. In addition, the early local innate response to SARS-CoV-2 infection, which is facilitated by TI, may support rapid virus control, keeping systemic inflammation low and enabling recovery. In contrast, absence of TI may increase the risk of hyperinflammation and severe complications associated with viremia (7).

In the present study we show for the first time that intravesical BCG also induces innate immune responses against SARS-CoV-2. The major new findings of the present study are: (i) BCG did not induce B cell or T cell-dependent adaptive immunity against SARS-CoV-2; (ii) BCG-treated NMIBC patients expressed key cytokines of trained immunity at the mRNA and protein levels after stimulation with SARS-CoV-2; (iii) ATAC sequencing revealed that repeated BCG instillations cause an increased accessibility of important TI genes (*TLR2*, *IGF1R*, *AKT1, MTOR, MAPK14, HSP90AA1*). Collectively, intravesical BCG indeed causes TI, which is the basis of innate immunity against SARS-CoV-2 (**Figure 6**). An important aspect of our findings is that the local administration of BCG into the bladder also promotes systemic immunity after at least six BCG instillations, reflected by the increased responsiveness of circulating immune cells. In general, epigenetic and metabolic reprogramming leading to TI needs some time. With regard to BCG instillations in NMIBC patients, repetitive administrations are required to observe an increase in innate immunity. In our study, TI cytokines appeared after 6 weeks. This is in accordance with recent work proposing TI as a molecular mechanism for BCG immunotherapy in bladder cancer (11). Although van Puffelen and coworkers assessed numbers of circulating leukocytes in whole blood early, i.e. one week after BCG1, changes in cytokine production were also not reported before BCG6 (11). The authors argue that late time points of cytokine measurements are most informative for TI (42).

In accordance with our current findings, a recent study has also shown that intravesical instillations of BCG induce TI at a systemic level (11). While these authors observed BCG-induced immunity against pneumonia and common cold in bladder cancer patients (11), we report for the first time on BCG-driven innate immunity against SARS-CoV-2. Of note, all blood samples analyzed in our study have been collected from NMIBC patients in the pre-Covid-19 era (2014-2015), most likely excluding the possibility of previous contact with SARS-CoV-2, which is also supported by the complete absence of RBD specific antibodies in these individuals. Accordingly, SARS-CoV-2-induced pro-inflammatory cytokine production could not be detected in control samples collected before BCG initiation. On the contrary, repetitive BCG administration was required to observe SARS-CoV-2-induced production of TI cytokines. Moreover, we could not detect adaptive immune responses (neutralizing antibodies, T cells) against SARS-CoV-2 excluding the existence of virus-specific memory lymphocytes.

One limitation of the study is the small number of patients, which is due to the fact that we used samples from a pre-COVID-19 era. Moreover, our data cannot be generalized as different BCG strains with immune-reactive differences in elicited immune responses are used in clinical practice (43). However, the BCG strain RIVM which was used in this study is mainly adopted across all EU countries.

Finally, our data reinforce the fact that adjustments to BCG induction and maintenance schedules, as recommended at the early onset of the COVID-19 pandemic (3, 4), are not necessary in clinical practice. Although our data support the development of BCG-induced TI, we did not have the opportunity to study TI-mediated protection against SARS-CoV-2 infection. However, it should be emphasized that research leading to the discovery of TI has been prompted by epidemiological studies showing that BCG vaccination at birth results in reduced child mortality and by subsequent randomized controlled trials demonstrating, that the beneficial effects of BCG were mainly due to protection against neonatal sepsis and respiratory infections (19). The BCG-induced TI observed in our study that includes responsiveness against SARS-CoV-2 in NMIBC patients is thus likely to mediate protection against Covid-19. Thus, every effort should be made to administer full BCG maintenance in high-risk NMIBC patients as recommended even during the pandemic period. Moreover, BCG-induced trained immunity could be one of the most important mechanisms mediating efficacy to BCG in bladder cancer (11, 44). Thus, further trials are urgently needed to evaluate how trained immunity influences the antitumor immune responses in BCG-treated NMIBC patients, improving personalized medicine in NMIBC (11, 44).

## Conclusions

Here we present the first study showing that repetitive intravesical BCG instillations in high- risk NMIBC also induces innate immune responsiveness against SARS-CoV-2 via mechanisms of TI. The increased fitness of the innate immune system that is induced by repetitive BCG administrations may be important in the context of various therapeutic and preventive strategies.

## Data availability statement

The datasets presented in this study can be found in online repositories. The names of the repository/repositories and accession number(s) can be found below: https://zenodo.org/record/7825968.

## Ethics statement

The studies involving human participants were reviewed and approved by AN2014-0121; 336/4.3. The patients/participants provided their written informed consent to participate in this study.

## Author contributions

RP, GD, JK, MT and WP processed and analyzed data and wrote the manuscript. RP, LM, DD, EL, AM, BP, FS, WK, MM, FG and JT were involved in patient recruiting, collection processing and analysis of samples and clinical data. GD, JK and WP performed and analyzed cell culture, neutralization and ELISpot assays. HH performed and analyzed ATAC sequencing. GD and JK performed experiments and analyzed data. MT, WP, MM, LM, DD, AM and FS helped with manuscript preparation, provided expertise and discussed the data. MT and WP were responsible for supervision. All authors contributed to the article and approved the submitted version.
