# Supplementary material for: Intravesical BCG in bladder cancer induces innate immune responses against SARS-CoV-2
Source: Front Immunol. 2023 Jul 13;14:1202157. doi: 10.3389/fimmu.2023.1202157 (PMC10374029; doi:10.3389/fimmu.2023.1202157)
Supplement: Supplementary file 1 [file Image_1.pdf]

Supplementary material

Figure S1

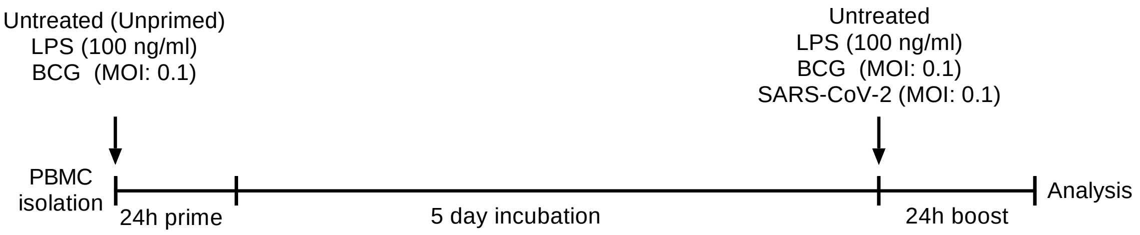

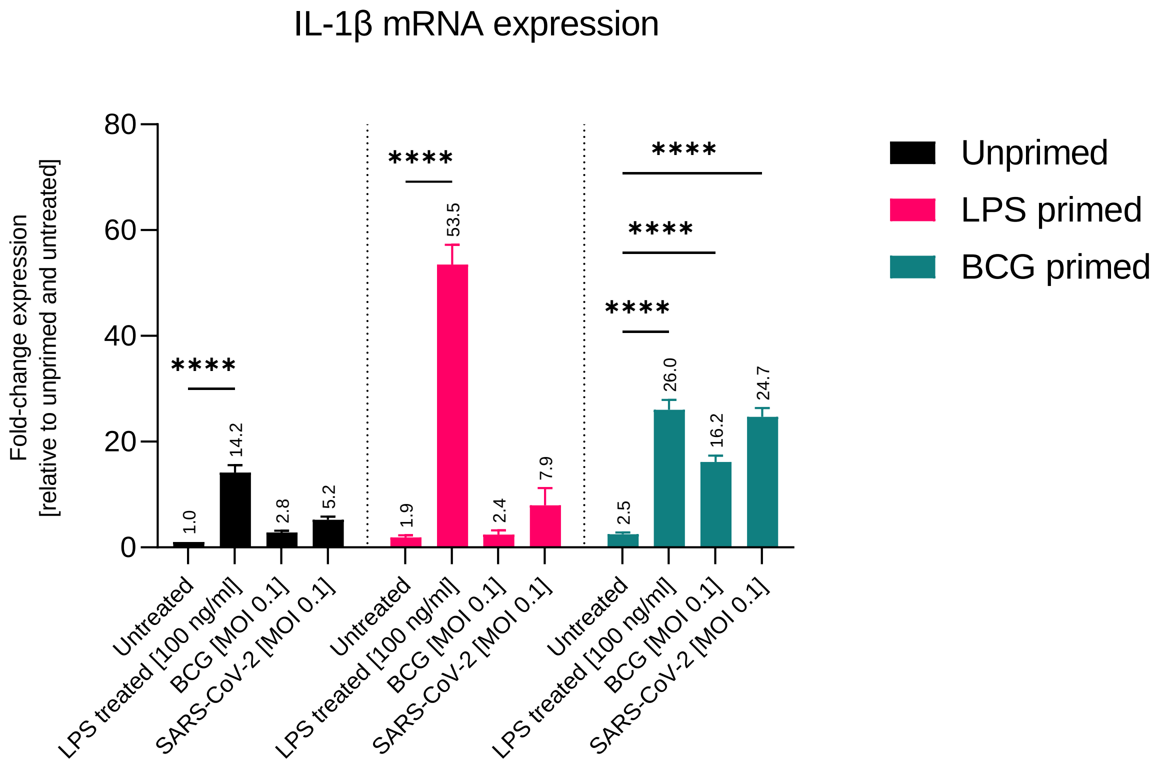

Figure S1. LPS and BCG induced trained immunity in healthy human PBMCs. Interleukin 1β mRNA expression in PBMCs from healthy human blood donations without prior BCG treatment. Cells remained untreated or primed for 24h with LPS (100 ng/ml) or BCG (MOI: 0.1) and incubated for 5 days. Following incubation, cells were either left untreated or re-stimulated with LPS (100 ng/ml), BCG (MOI: 0.1) or SARS-CoV-2 (MOI:0.1).

Data is presented as fold-change expression relative to untreated and unprimed control group (mean with SEM; ****$p < .0001$). Time points: 1-2 weeks/early, 3-4 weeks/mid and 6-12 week-interval/late during BCG.
